# “Too Big To Ignore”: A feasibility analysis of detecting fishing events in Gabonese small-scale fisheries

**DOI:** 10.1371/journal.pone.0234091

**Published:** 2020-06-10

**Authors:** Floriane Cardiec, Sophie Bertrand, Matthew J. Witt, Kristian Metcalfe, Brendan J. Godley, Catherine McClellan, Raul Vilela, Richard J. Parnell, François le Loc’h

**Affiliations:** 1 IRD, Univ Brest, CNRS, Ifremer, LEMAR, Plouzané, France; 2 Wildlife Conservation Society, Gabon Program, Libreville, Gabon; 3 IRD, UMR Marbec, Univ Montpelier, CNRS, Ifremer, Sète, France; 4 Hatherly Laboratories, College of Life and Environmental Sciences, University of Exeter, Exeter, United Kingdom; 5 Centre for Ecology & Conservation, College of Life and Environmental Sciences, University of Exeter, Penryn Campus, Penryn, United Kingdom; 6 ONG Manga, Akanda, Gabon; Tanzania Fisheries Research Institute, UNITED REPUBLIC OF TANZANIA

## Abstract

In many developing countries, small-scale fisheries provide employment and important food security for local populations. To support resource management, the description of the spatiotemporal extent of fisheries is necessary, but often poorly understood due to the diffuse nature of effort, operated from numerous small wooden vessels. Here, in Gabon, Central Africa, we applied Hidden Markov Models to detect fishing patterns in seven different fisheries (with different gears) from GPS data. Models were compared to information collected by on-board observers (7 trips) and, at a larger scale, to a visual interpretation method (99 trips). Models utilizing different sampling resolutions of GPS acquisition were also tested. Model prediction accuracy was high with GPS data sampling rates up to three minutes apart. The minor loss of accuracy linked to model classification is largely compensated by the savings in time required for analysis, especially in a context of nations or organizations with limited resources. This method could be applied to larger datasets at a national or international scale to identify and more adequately manage fishing effort.

## Introduction

In many developing countries, small-scale fisheries are the mainstay of the fisheries sector [1]. Approximately 50 million people worldwide are employed directly in fishing, of which 22 million (44%) are associated with small-scale fisheries [2]. This sector, therefore, makes a considerable contribution to local and national economies due to its important role in food security, employment, and as a potential route to poverty alleviation. It has been demonstrated that in comparison to large-scale industrial fishing, small-scale fisheries provide more employment, have lower production costs, produce fewer discards [3], and may be more likely to promote the sustainable use of marine species, as they respond dynamically to resource fluctuations [4]. Despite this, small-scale fisheries are often under-studied in comparison to large-scale industrial fleets [5–8].

Among the main challenges to describing and quantifying the spatial distribution and pressures associated with small-scale fisheries are: (1) a lack of resources directed towards data collection; (2) the distant and dispersed nature of fisheries (e.g. remote landing sites); (3) a scarcity of fine-scale spatial data on fishing effort; and (4) a lack of stakeholder engagement and participation in data collection [9,10]. This paucity of information, together with the complex socio-economic conditions of communities involved in this sector can have two important and negative consequences. First, the lack of data can lead to an underestimation of fishing effort and hence overexploited fisheries [7]. Second, it can marginalize communities from decision-making processes and so lead to increased conflict, particularly with government agencies [9].

As fishing effort varies with location and season, it is important to design spatial management plans for stocks and fisheries [11–14]. However, creating a spatiotemporal footprint of coastal fisheries is often more challenging than for industrial fleets that are frequently tracked using Vessel Monitoring Systems (VMS) or Automatic Identification Systems (AIS) [15–17]. As such, the tracking of small-scale fleets tends to require the use of novel approaches. A number of such approaches have been developed including interviews [18], participatory mapping [19], at-sea transects [20], modelling using generalized behavior rules such as distance from the shore and fishing depths [21], number of boats and coastal populations [22] or a combination of these methods [23–26].

Recent advances in remote monitoring technologies, have led to an increase in the use of GPS tracking devices to study small-scale fisheries [27–30]. Despite requiring more frequent intervention for downloading data and for servicing, small GPS tracking devices are capable of collecting similar data to VMS and AIS systems and are more appropriate for small vessels without dedicated electrical systems. Several studies have attempted to understand fishing behavior using VMS data, by separating fishing activity from other activities (cruising, searching, transiting, hauling) based mostly on simple thresholds on speed [15,16,31–34] and sometimes combined with turning angles [12,35]. In the last decade, more refined approaches have been investigated using Generalized Linear Models [36], Gaussian Mixture Models [37,38], Random Forest [39] and, Neural Networks [40–42]. In the case of data with temporal dependence, a generative model such as Hidden Markov Model (HMM) is appropriate. When part of the data is available for training [43] and sampling is done with a regular time interval [36], HMMs are the generally preferred methodology [44–50] and have been shown to provide accurate detections of fishing patterns, especially when speed and turning angle are chosen as variables [38,51].

Small-scale fisheries in Gabon are a perfect illustration of the gap between the importance of the sector and the level of active research and management. The most recent published statistics from 2014 indicate that while industrial fisheries produced 7,026 tons, small-scale marine fisheries produce 18,076 tons (i.e. 72% of the national production [52]). With approximately 1,200 wooden boats along the coast, Gabonese small-scale fisheries are characterized by a great diversity of fishing gears and techniques, practiced by a variety of different communities [53]. While VMS is mandatory for industrial vessels in Gabon, little has been achieved with regard to spatially mapping of small-scale fisheries. In this experimental study, GPS trackers were used to address this knowledge gap. As technology develops and management issues arise, deployment of GPS devices in small-scale fisheries is anticipated to increase. With no electrical power on most small fishing boats, devices are still limited by battery life and/or data storage capacity. Given these limitations, it is important to gain a better understanding of the potential artifacts, limitations and benefits of differing sampling strategies resulting from working on these often geographically remote, exposed and more logistically challenging fishing environments.

Using small-scale fisheries in Northern Gabon, we examined two approaches to processing tracking data (expert interpretation of fishing tracks and the use of Hidden Markov Models) and investigated the most effective sampling strategies to make use of low-cost tracking solutions for this fleet.

## Materials and methods

### GPS data collection and pre-processing

**Fig 1:**
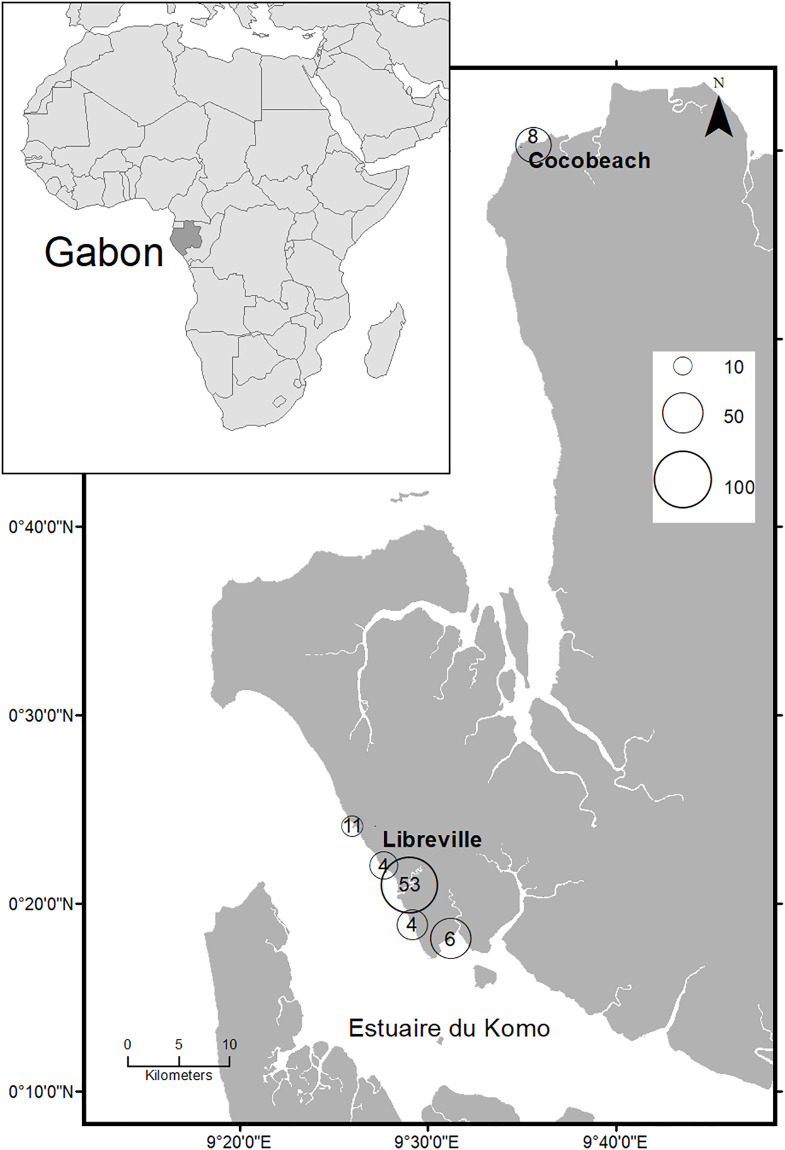
Map of the North of Gabon showing the size of the fleet and the number of fishing trips sampled by landing site. Size of circles represents estimated number of boats by landing site and number inside is the count of fishing trips sampled in this location.

Between February 2013 and September 2014, GPS devices (Model 1: Garmin Etrex 20, n = 31; Model 2: Mobile Action GT600 motion sensing tracker, n = 6) were deployed on 99 individual fishing trips undertaken by small-scale motorized fishing boats from 6 landing sites in Libreville and Cocobeach (Fig 1). Devices were programmed to record positions at high frequency (5-second intervals), to collect fine scale spatiotemporal data and allow subsequent degradation of the time interval during analysis. Small-scale fisheries are defined here according to Gabonese regulations, as boats that require little financial or technical investment, and which principally rely upon manual manipulation of fishing gear (MEFPE 1994; République Gabonaise 2005). Fishing gears utilized varied within and between the communities using each landing sites. Each trip was, therefore, assigned to one of seven fishing gears: (1) surface drifting gillnet, (2) bottom gillnet, (3) purse seine, (4) circling gillnet for sardines, (5) circling gillnet for mullets, (6) longline and (7) handline.

The average deployment duration of each GPS unit ranged between 1 and 10 days before needing to be recharged and/or replaced with a new unit. The number of individual fishing trips recorded for each gear ranged between 8 and 32 (mean±SD number of trips recorded per gear: 14 ± 8; n = 7 gears). Once each GPS unit was retrieved, the recorded data were downloaded and processed to remove possible erroneous location data, which included: (1) removing pre- and post-deployment locations associated with travel to and from the landing sites; (2) removing locations that were within 3 km of landing sites as these are associated with transiting and are areas where fishing is prohibited; and (3) applying a speed filter to remove locations with a speed > 50 km.h^-1^. This threshold was based on maximal speed of 40 HP engines typically used by small-scale fisheries. In a time series of locations, time interval standardized data are a prerequisite for Hidden Markov Models, therefore, GPS data for each fishing trip were standardized to 5-second intervals to avoid time lag and to eliminate missing locations from poor satellite coverage using speed-based linear interpolation. After processing, the resulting dataset comprised of 99 tracks, and 818,747 vessel locations (longitude and latitude decimal degrees, WGS84). To gain an insight into the general operating behavior of small-scale fisheries in the region, information on total trip duration and total distance were calculated and summarized for each individual gear type. Data were processed in R using *adehabitatLT*, *rgdal*, *geosphere* packages in R [54,55].

### Characterizing behavioral fishing states

We investigated two approaches to identify fishing behavior within GPS tracking data (n = 99), using data from on-board observers (n = 7) regarding fisher behavioral changes as reference: (1) visual interpretation based on several characteristics of fishing behavior determined by on-board observers and (2) application of Hidden Markov Model to GPS tracking data. Due to the low sample of trips with observers on-board, the visual inspection was employed as the truth against which to assess the accuracy of HMM.

#### On-board observers

For each fishing gear (n = 7), an observer accompanied a single fishing trip recording fishing practices and behaviors such as: time of departure, start and end time of gear deployment, start and end time of gear retrieval (e.g. hauling), and time of return to the landing site.

From these data and associated GPS tracks, the unique fishing “signature” for each fishing gear was characterized (i.e. typical shape, speed and turning angle; Fig A in S1 File). Artisanal fishers in Gabon transit to fishing grounds upon which the crew actively search for signs at the sea surface before deploying their gear. In other studies, each fishing trip could be divided into distinct behaviors: transiting, searching, setting, fishing and hauling [44]. In our study, as the typical engine used is relatively low-powered (i.e. 40 HP), vessel speed is maintained during the searching phase. For all fishing operations, there is a phase of gear deployment (i.e. fishing commences). For the following gear types: longline, drifting and bottom gillnet, fishers wait with the gear while it soaks and fish are caught. The gear is then retrieved from the water (i.e. fishing ceases) and a transiting phase follows as boats return to the port, or to commence further fishing operations.

#### Visual interpretation

These signatures were used to identify fishing operations for remaining tracks that did not have on-board observers (n = 99 trips). This process involved 3 experts (including FC) who have > 10 years of experience working on artisanal fisheries in Gabon and so have a detailed understanding of fishing practices and behaviors displayed by distinct communities. Each expert analyzed tracks from communities they know the best and, therefore, each track has been analyzed only by one expert. They visualized the track shape and pattern along with information on speeds to distinguish each individual fishing operation. This method based on expert knowledge has already been used in a previous study [43]. Each location in a track was subsequently classified as either ‘fishing’ or ‘non-fishing’. When vessel speed was less than 7 km.h^-1^ and the typical shape of a fishing operation was observed, the start and end time of the fishing operation were recorded.

#### Hidden Markov Model theory

To identify fishing activity from track metrics, a Hidden Markov Model was applied. HMMs are commonly used as discrete time-series models to represent probabilities of hidden states [51,56–60] and are increasingly being employed to analyze fishing vessel movements [43–47,61].

According to [62], a HMM is characterized by:

1. N, the number of states in the model. In this case study, those states correspond to fisher behavior, for instance being at anchor, traveling, fishing, etc. The individual state is noted S = {S_1_, S_2_, …, S_N_} and the state at time t is q_t_.
2. M, the number of distinct observations. Here, it is the number of segments (steps) between two successive positions in a track. The individual observation is noted as V = {V_1_, V_2_, …, V_M_}
3. The state transition probability distribution A = {a_ij_} where
  $$\begin{array}{cc} {\text{a}}_{\text{ij}}=\text{P}[{\text{q}}_{\text{t}+1}={\text{S}}_{\text{j}}|{\text{q}}_{\text{t}}={\text{S}}_{\text{i}}), & 1\leq \text{i},\;\text{j}\leq \text{N} \end{array}$$
  In our case, it’s the probability to switch, for instance, from a fishing activity to transiting between t and t+1. It’s one of the main characteristics of HMM that q_t+1_ depends on q_t_ but is completely independent of the previous state q_t-1_.
4. The observation probability distribution in state j, B = B {b_j_(k)} where
  $$\begin{array}{cc} {\text{B}}_{\text{j}}\left(\text{k}\right)=\text{P}[{\text{v}}_{\text{kt}}|{\text{q}}_{\text{t}}={\text{S}}_{\text{j}}], & 1\leq \text{j}\leq \text{N}\\ & 1\leq \text{k}\leq \text{M} \end{array}$$
  Here, it is the probability for a segment V_k_ to be in a specific state S_j_.
5. The initial state distribution π = {π_i_} where
  $$\begin{array}{cc} \pi_{\text{i}}=\text{P}[\text{q}1=\text{Si}], & 1\leq \text{i}\;\leq \text{N} \end{array}$$
  The initial state corresponds to the state the first observation takes.

#### Choice of observed variables, number of states and initial parameter values

To determine fishing activity within tracking data, speed is commonly used [36] as well as turning angles [37,39,49], as these characteristics of movement change with fisher activity. When transiting to a fishing ground, the GPS track tends to produce a straighter trajectory with a higher speed as opposed to when it is fishing, when the trajectory tends to be more sinuous with a lower speed in artisanal fisheries in Gabon (as seen in Fig B in S1 File). Distance between each location (step) and turning angles were used as observed variables in the model. The choice of the number of states for the model is crucial and depends on the study system [63]. To choose the number of states, the distribution of speeds for each gear was observed and integrated with existing knowledge about fishing techniques.

In HMMs, seed values for the model parameters need to be specified. In this case, data from on-board observers were used to provide initial parameter values. Step lengths followed a gamma distribution, and angles a Von Mises distribution [64]. For each gear type, mean and standard deviation of step lengths and concentration of angles were calculated for each state (fishing and non-fishing), to determine initial parameter values.

#### HMM fitting

The R package *MoveHMM* was used to determine fishing and non-fishing states from the GPS tracking data [57,65]. A Hidden Markov Model was fitted for each gear type, using the forward algorithm [65]. To determine the most probable state sequence, a Viterbi algorithm was used. Pseudo-residual distributions from the HMM process were visually inspected to confirm normal distribution.

### Method performance evaluation

Confusion matrices were used to determine the performance of the HMM and visual interpretation approaches. Using the confusion matrix, several performance measures were calculated, these included: (1) sensitivity, which is the probability that a test will indicate 'fishing' among those which were actually fishing; (2) specificity which is the probability that a test will indicate 'non-fishing' among those which were actually not fishing; (3) fishing prediction which indicates the probability of true fishing positions among fishing positions detected by the model; and (4) non-fishing prediction which indicates the probability of true non-fishing positions among non-fishing positions detected by the model [66].

For trips with observers on-board (n = 7), observed data on the occurrence of fishing activity were compared to fishing detected during expert interpretation and from the HMM process. Visual interpretation and HMM approaches were also compared. Since for the rest of tracks (n = 99), fishing trips did not have an observer on-board, the visual interpretation was used as the truth against which to assess the accuracy of HMM. To test the utility of this approach on datasets with different temporal sampling resolutions, the HMM was fitted to data regularized at different time intervals (i.e. 10, 20, 30, 40, 50 and 60 seconds, and each of 2 to 10 minutes at 60-second intervals). Ten minutes was the maximum time step tested as it was typically the shortest duration of a fishing operation, especially for handlines.

To assess the most efficient method for detecting fishing activity within GPS tracking data (i.e. minimum analytical time), three time-based metrics were calculated: (1) the mean duration of a fishing trip hosting an on-board observer, (2) the mean duration required to visually inspect tracking data during the expert interpretation process, and (3) the mean duration required to complete the execution of the HMM.

## Results

### Cleaned data characteristics

**Fig 2:**
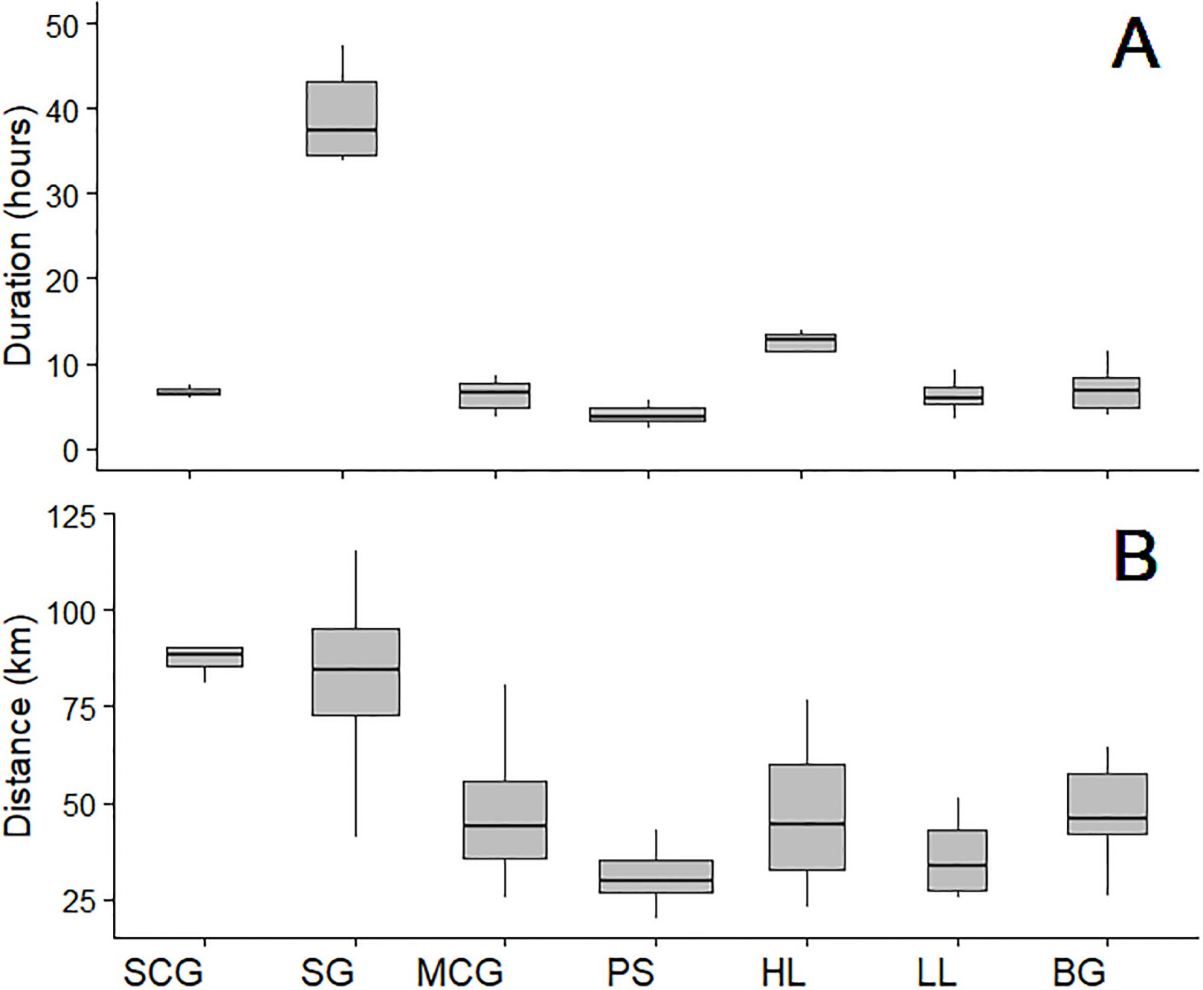
Boxplots of trip duration (A) and distance covered (B) for n trips by gear type. SCG = Sardine Circling Gillnet (n = 10), SG = Surface Drifting Gillnet (n = 14), MCG = Mullet Circling Gillnet (n = 12), PS = Purse Seine (n = 32), HL = Handline (n = 12), LL = Longline (n = 8) and BG = Bottom Gillnet (n = 11).

Fishing characteristics (e.g. trip duration and distance covered) varied with fishing gear type (Fig 2). Some fishing techniques occurred during a single day such as sardine circling gillnets, mullet circling gillnets and purse seines; the minimum trip duration was 02:19 hours for purse seins. Other techniques, however, extended through day and night (longlines, bottom gillnets) or even several days per trip (surface drifting gillnets, handlines); the maximum trip duration was 76:11 hours for surface drifting gillnets.

Variation among gear types was also observed with the total trip distance, with up to 165 km covered for surface drifting gillnet trips, or with more coastal fishing methods like purse seines where the minimum trip distance was 26 km.

### Behavioral states

**Fig 3:**
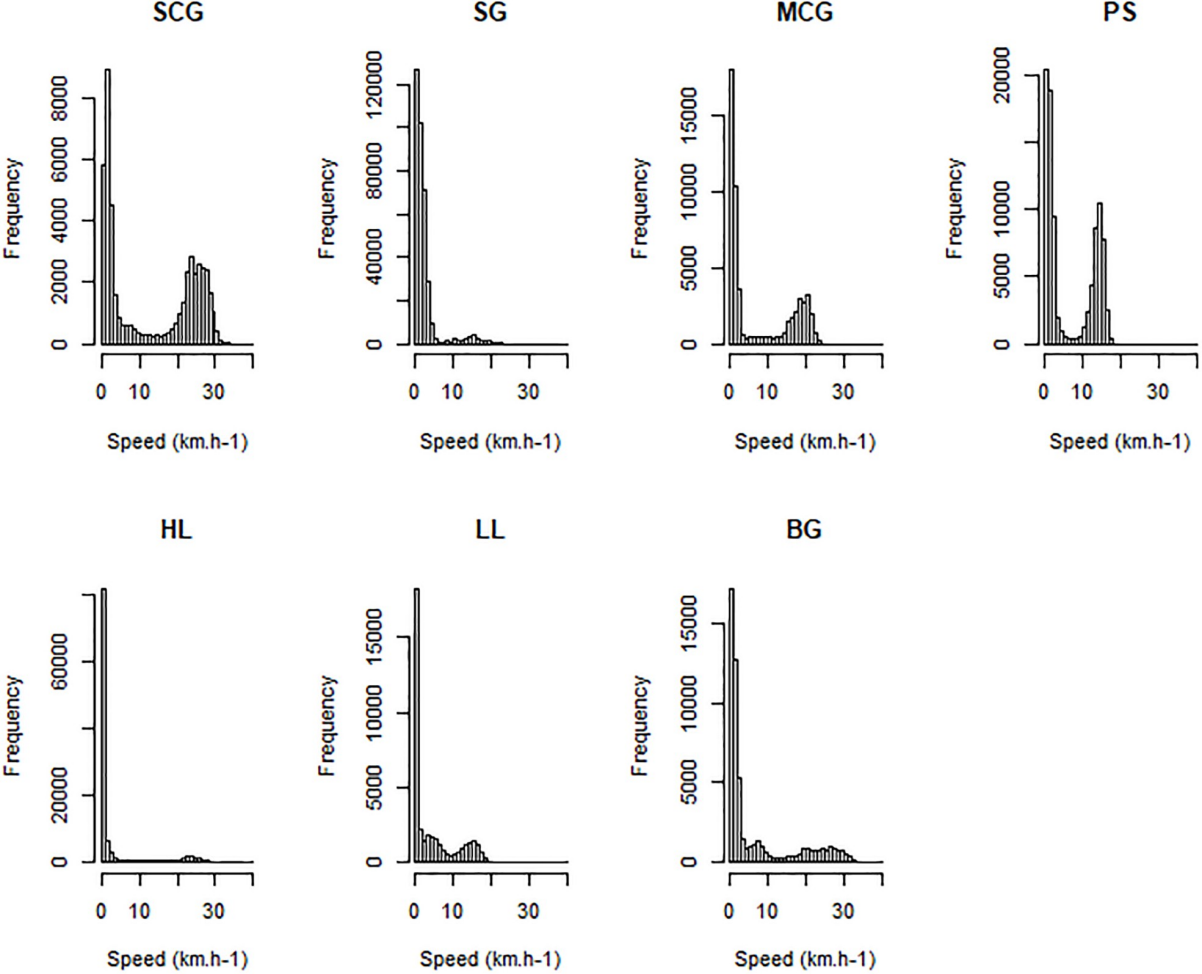
Distribution of vessel speed for each gear type. SCG = Sardine Circling Gillnet, SG = Surface Drifting Gillnet, MCG = Mullet Circling Gillnet, PS = Purse Seine, HL = Handline, LL = Longline and BG = Bottom Gillnet.

In this study, behavioral states were simplified to “fishing” and “non-fishing” activities. Indeed, speed distributions for each gear type (Fig 3) revealed a bimodal distribution and, as such, a two states HMM modeling process was considered the most parsimonious approach.

**Fig 4:**
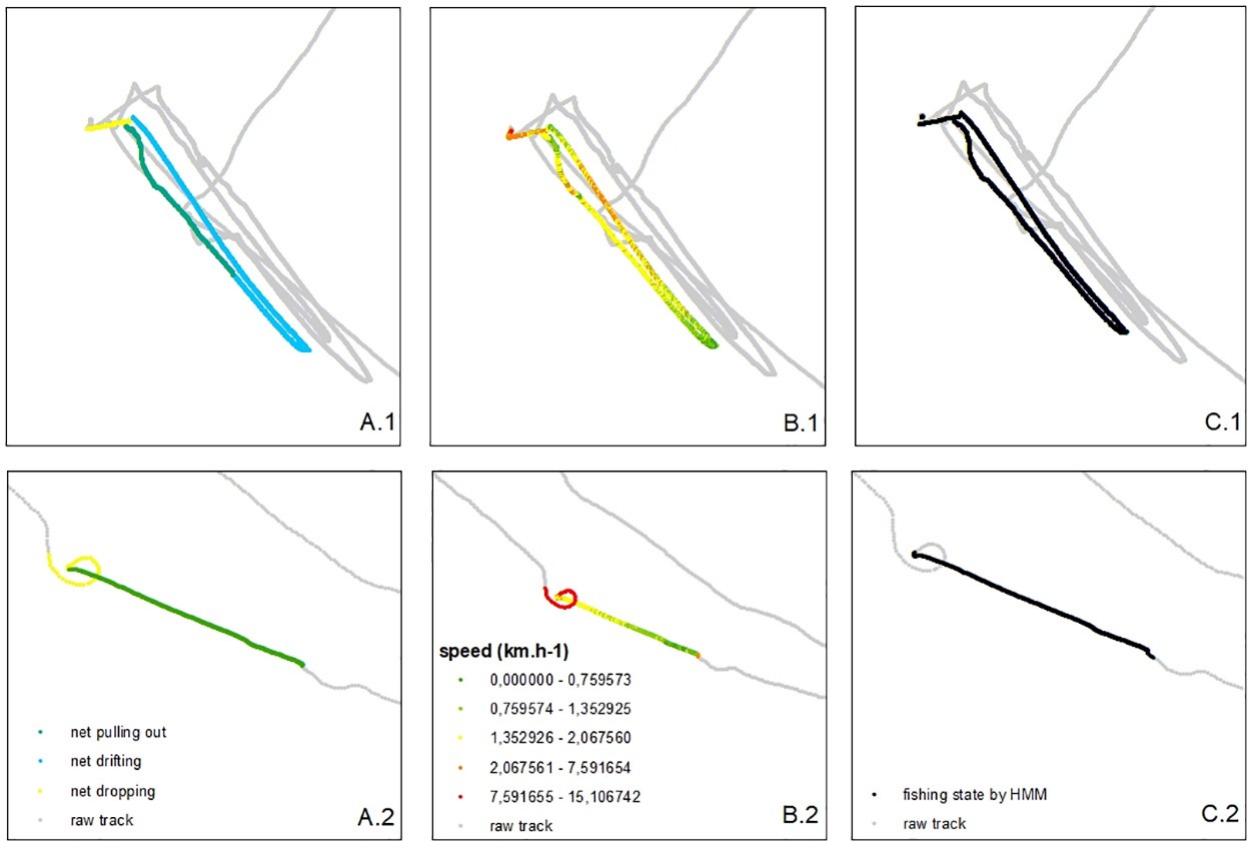
Mapping of 2 different methods used to identify a fishing event on a fishing trip (B: Visual interpretation, C: HMM) compared to observed data (A: On-board observer) for two different gear types (1: Surface drifting gillnet, 2: Purse seine).

For each gear type, one track was split into different behaviors thanks to an observer on-board. These observed data were used to calculate initial values to fit the HMM for each gear type (Table A in S1 File), and the shape of a fishing operation within the GPS track was memorized as a “fishing signature” (Fig A in S1 File), which was then used to recognize fishing patterns (combined with vessel speed) in the visual interpretation method (Fig 4).

Based upon the confusion matrix, accuracy was assessed between observed data (when an observer was on-board) and the visual interpretation classification (Table 1). Median accuracy was 0.95 and, values were higher for fishing detection (sensitivity) and fishing prediction than for non-fishing detection (specificity) and non-fishing prediction. Sardine circling gillnets and handlines experienced the lowest accuracy (0.90). When comparing observed data with HMM predictions (Table 2), the median accuracy was 0.80. The fishing prediction was better than the non-fishing prediction. The sardine circling gillnets produced the lowest accuracy (0.72), with a weak specificity (0.59), followed by longlines with the 0.75 accuracy due to the lowest specificity (0.49). Other gear types produced accuracies greater than 0.8. Comparison between visual interpretation and HMM predictions (Table 3) produced a median accuracy of 0.8, whereby the lowest accuracies were for longlines (0.71), sardine circling gillnets (0.79) and mullet circling gillnets (0.79).

**Table 1:** Performance measures between on-board observers and visual interpretation for each gear type.

	Accuracy	Sensitivity	Specificity	F prediction	NF prediction
Sardine circling gillnet	0.90	0.98	0.87	0.77	0.99
Surface drifting gillnet	0.98	0.99	0.85	0.98	0.89
Mullet circling gillnet	0.96	0.96	0.95	0.96	0.95
Purse seine	0.95	0.97	0.92	0.97	0.92
Handline	0.90	0.95	0.46	0.94	0.48
Longline	0.95	0.94	0.97	0.97	0.93
Bottom gillnet	0.96	1.00	0.63	0.96	1.00
MEDIAN (MAD)	0.95 (±0.00)	0.97 (±0.02)	0.87 (±0.08)	0.96 (±0.01)	0.93 (±0.04)

Analysis was conducted on the seven trips with observers on-board, with data collected on-board taken as the truth. The sensitivity expresses the fishing detection and the specificity, the non-fishing detection. (F = fishing, NF = non-fishing, MAD = Median Absolute Deviation).

**Table 2:** Performance measures between on-board observers and HMMs.

	Accuracy	Sensitivity	Specificity	F prediction	NF prediction
Sardine circling gillnet	0.72	1.00	0.59	0.52	1.00
Surface drifting gillnet	0.80	0.79	0.92	0.99	0.32
Mullet circling gillnet	0.80	0.99	0.51	0.75	0.98
Purse seine	0.97	0.97	0.97	0.99	0.92
Handline	0.91	0.90	1.00	1.00	0.52
Longline	0.75	0.98	0.49	0.69	0.96
Bottom gillnet	0.82	0.81	0.89	0.98	0.37
MEDIAN (MAD)	0.80 (±0.05)	0.97 (±0.03)	0.89 (±0.11)	0.98 (±0.02)	0.92 (±0.08)

Analysis was conducted on the seven trips with observers on-board, with data collected on-board taken as the truth. The sensitivity expresses the fishing detection and the specificity, the non-fishing detection. (F = fishing, NF = non-fishing, MAD = Median Absolute Deviation).

**Table 3:** Performance measures between visual interpretation and HMMs.

	Accuracy	Sensitivity	Specificity	F prediction	NF prediction
Sardine circling gillnet	0.79	0.98	0.66	0.65	0.98
Surface drifting gillnet	0.80	0.80	0.91	0.99	0.30
Mullet circling gillnet	0.79	0.99	0.50	0.75	0.97
Purse seine	0.95	0.96	0.95	0.98	0.89
Handline	0.81	0.84	0.51	0.94	0.25
Longline	0.71	0.96	0.45	0.65	0.92
Bottom gillnet	0.80	0.80	1.00	1.00	0.26
MEDIAN (MAD)	0.80 (±0.01)	0.96 (±0.03)	0.66 (±0.22)	0.94 (±0.06)	0.89 (±0.09)

Analysis was conducted on the seven trips with observers on-board, with visual interpretation considered as the truth. The sensitivity expresses the fishing detection and the specificity, the non-fishing detection. (F = fishing, NF = non-fishing, MAD = Median Absolute Deviation).

### Modelling *versus* visual interpretation

When HMM was applied to all the data and compared to the visual interpretation method considered as truth, the median accuracy was 0.75, and performance varied depending on the gear type (Table 4). The lowest accuracy (0.69) was obtained for surface drifting gillnets. The best accuracy (0.85) was achieved by the purse seines. The median sensitivity was higher than the median specificity and the median non-fishing prediction was higher than the median fishing prediction. The percentage of time spent fishing during a fishing trip was also compared between methods. Fishing time was greater for each gear type when calculated from HMM results than for visual interpreted states, except for longlines and bottom gillnets (Table 4).

**Table 4:** Performance measures of the HMM compared to visual interpreted states for all tracks (n = 99) and percentage of trip duration spent fishing (F = fishing, NF = non-fishing).

	Performance measures					% of trip fishing	
	Accuracy	Sensitivity	Specificity	F prediction	NF prediction	visual interpretation	HMM
Sardine circling gillnet	0.78	0.96	0.66	0.66	0.96	41	59
Surface drifting gillnet	0.69	0.76	0.57	0.76	0.58	64	64
Mullet circling gillnet	0.75	0.95	0.57	0.67	0.92	48	68
Purse seine	0.85	0.94	0.77	0.79	0.94	47	57
Handline	0.74	0.85	0.43	0.80	0.51	73	77
Longline	0.75	0.96	0.43	0.72	0.88	60	38
Bottom gillnet	0.75	0.75	0.75	0.94	0.36	84	67
**Median**	**0.75**	**0.94**	**0.57**	**0.76**	**0.88**	**60**	**67**

**Fig 5:**
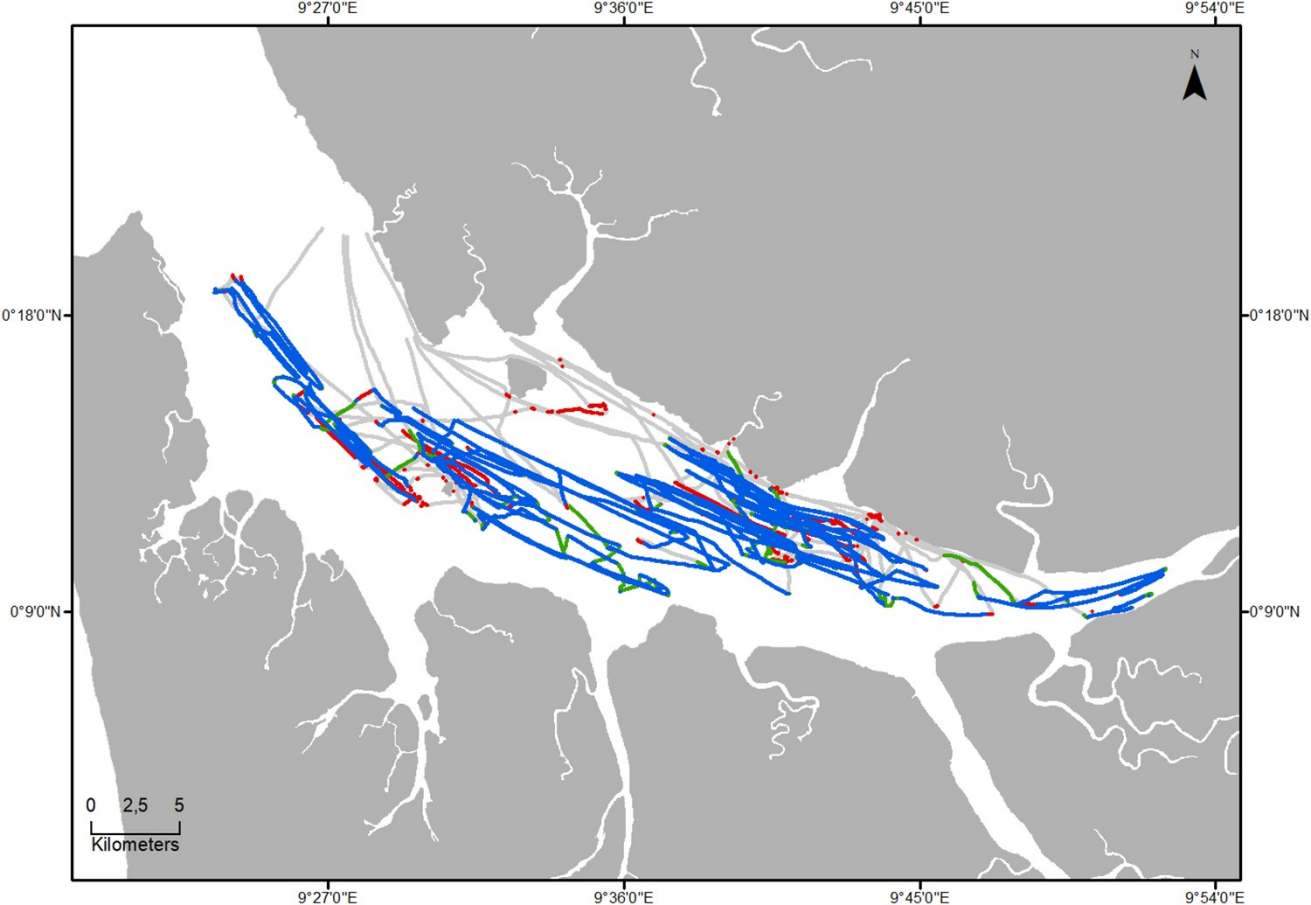
Map of all tracks for surface drifting gillnet gear type and fishing areas identified by visual interpretation and HMM methods (In grey: Both methods predicted non-fishing, in blue: Both methods predicted fishing, in red: Only HMM predicted fishing, in green: Only visual interpretation predicted fishing).

Surface drifting gillnets is the gear type that resulted in the lowest accuracy of all, and corresponds to 13 fishing trips and 372,378 points (Fig 5). Fishing and non-fishing state accuracies were 0.76 and 0.56, respectively. Results for other gears are presented in Fig B in S1 File.

### Performance measures with different sampling intervals

**Fig 6:**
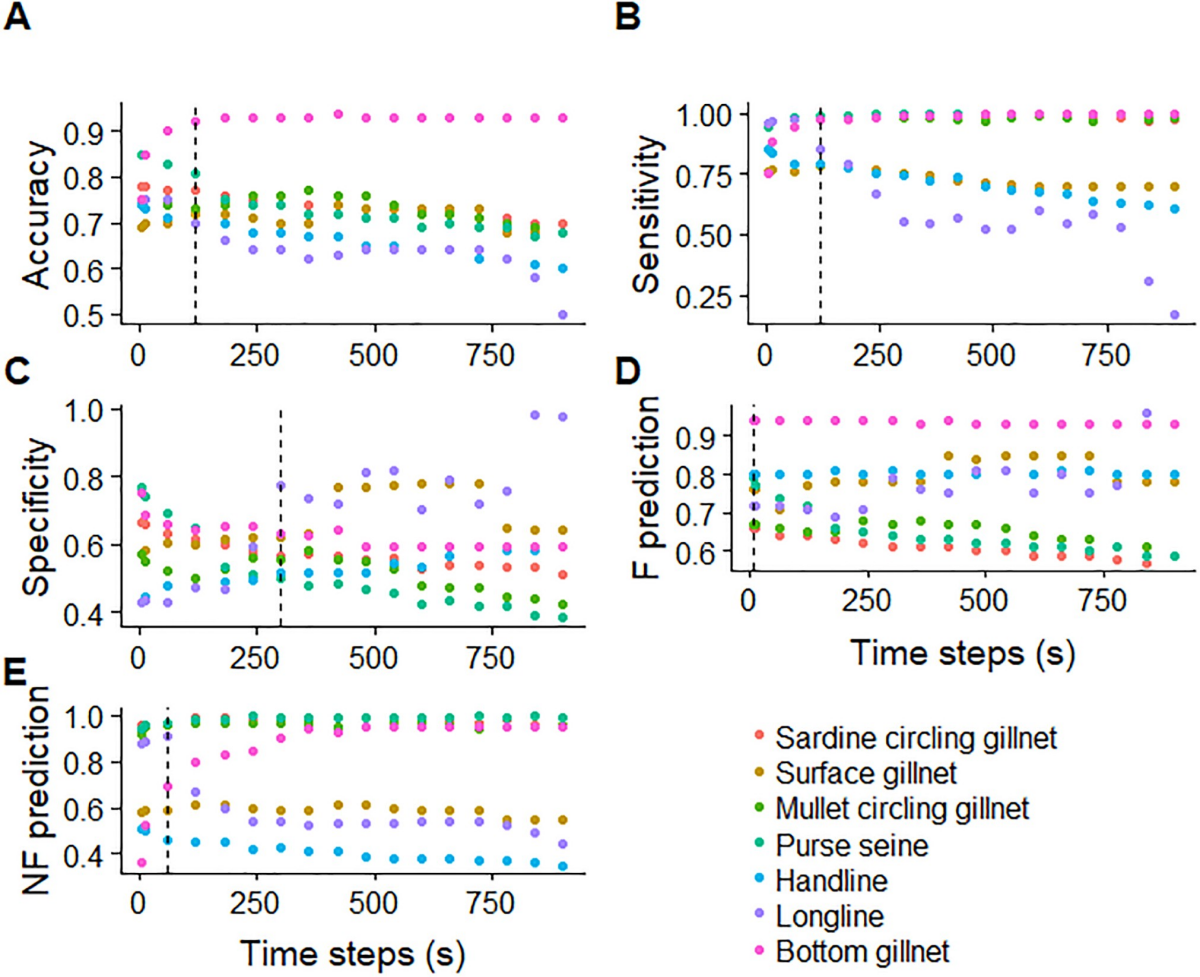
Accuracy, sensitivity, specificity, fishing prediction and non-fishing prediction across different time steps for each gear type. The dashed line shows the time step where values were highest. Values can be found in Table B in S1 File.

Performance metrics were examined for different sampling intervals, revealing substantial variation by gear type. For most gears, accuracy decreased with increasing time intervals between locations, with the exception of bottom gillnets, which increased (Fig 6A, Table B in S1 File). Accuracy was highest for bottom gillnets. Gears with consistently high sensitivity values (between 0.8 and 1), included: purse seines, sardine circling gillnets, bottom gillnets and mullet circling gillnets. Other gears produced lower sensitivity values that tended to decrease with increasing time steps, such as surface drifting gillnets, longlines and handlines (Fig 6B). Some gears showed decreasing specificity with increasing time steps, such as purse seines, mullet circling gillnets and sardine circling gillnets, while other gears such as handlines and surface drifting gillnets tended to show increasing specificity. Longlines and bottom gillnets showed varied specificity by first decreasing and then increasing (Fig 6C). The fishing prediction values for bottom gillnets were consistently high across time steps, while they tended to decline with time steps for purse seines, sardine circling gillnets and mullet circling gillnets. Other gears such as handline, longline and surface drifting gillnet seems to be stable (Fig 6D).

The optimal value for sampling frequency to achieve high accuracy and sensitivity was 120 seconds, 300 seconds for specificity, 5 seconds for fishing prediction, 60 seconds for non-fishing prediction (dashed lines in Fig 6).

### Comparison of methods

**Fig 7:**
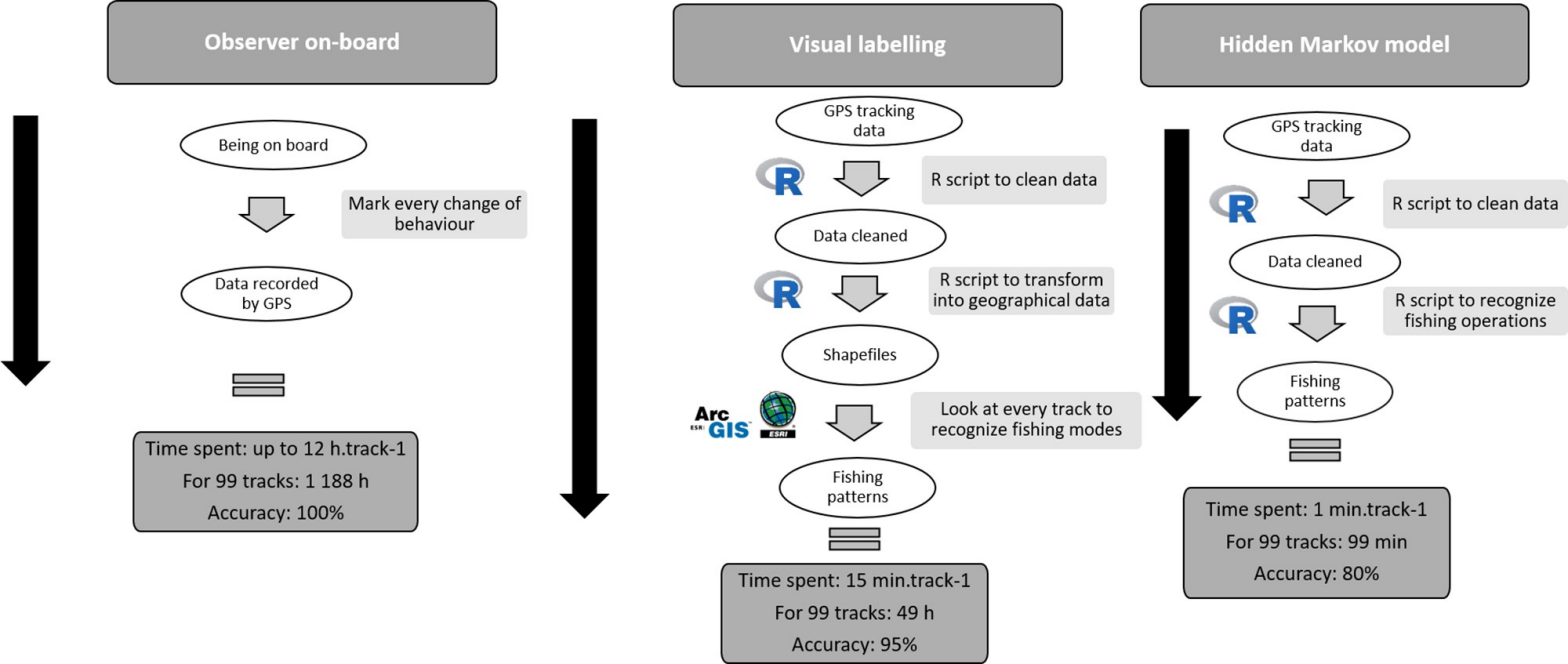
Stepwise overview of the processes of the two methods. Their time investment and estimated accuracy (%) are compared to having an on-board observer.

When the two methods were compared to having on-board observer data, regarding the time spent conducting analysis, the HMM method was the quickest, about 30 times less than the visual interpretation method and 720 times less than when an observer was on-board (Fig 7). The HMM method also required use of one software package (*R*), while the visual interpretation method required two software packages (*R* and a GIS). Median accuracy across gears was 95% for the visual interpretation method and 80% for the HMM method.

## Discussion

### Accuracy of methods

This study describes a way to identify and to map fishing behaviors for small-scale fisheries in Central Africa using Hidden Markov Models. We chose to develop models by gear type due to the large variation among their trip characteristics. Our visual interpretation approach had a high accuracy (0.95) when compared to data collected by on-board observers. The HMM results suggested that model accuracy was good enough to have an idea of actively-fished areas (median = 0.75) and proved to be about 30 times faster than visual interpretation of fishing states.

It was important to validate visual interpretation before comparing it to HMM. As having an on-board observer is expensive and not always possible, the visual interpretation approach has been accepted in similar studies [43,61]. Here, results show a high performance accuracy (0.95), but a lower performance for specificity (0.87), meaning that the visual interpretation method is not as good at detecting non-fishing behaviors. More precisely, when the comparisons were made by gear, non-fishing detection and prediction were low for handline fishing in this study. Indeed, when using this gear, fishermen slowed down to put the line in water, let it soak for a few minutes (10 min), moved the boat a short distance and put the line again in water. Those short moves between two fishing events were difficult to recognize visually. This weakness was, however, also found using HMM; the non-fishing prediction was low. The same issue was observed in bottom gillnet data, with the visual interpretation being weak at detecting non-fishing states. In those cases, transition between two fishing events could be classified as fishing by visual interpretation and by HMM.

Accuracy of the HMM became weaker when applied to a larger dataset but it is possible that the accuracy of the visual interpretation decreases also. To assess this tendency, more data from on-board observers would be necessary. Those data would also allow to have more precise model parameters but in a context of working with fishing communities, gaining trust takes time and observers are not always accepted. This is why the sample size was low and this should be addressed in future studies when trust is well established. Lower model performance accuracy occurred for fishing gears where fishermen let the gear soak longer in the water. In these cases, time was spent drifting and sometimes fishers left their gear in place and moved the boat to another area. In fact, the model was better at predicting fishing behaviors than it was at predicting non-fishing behaviors. During non-fishing patterns, fishermen could decrease speed and change direction for various reasons (engine stops, meeting with another boat, etc.). This was confirmed by comparing time spent fishing per trip among methods; the HMM over-detected fishing states (except for bottom gillnets). Still, accuracy was good enough to detect fishing areas, since high precision was not essential when gear length can be as much as 3 km long. A similar level of accuracy has been found when HMM was applied to data from VMS compared to those from on-board observers [51]. The model performance accuracy could be increased by adding covariates to the model, such as bathymetry and/or landing sites of origin, given that fishing methods depend on water depth and also on the fishing community. This should be explored in future studies.

### Understanding fishing techniques

Some fishing trips, such as those using surface drifting gillnets or longlines, typically result in a stay at-sea for several days, while fishers deploying other gears undertake fishing trips of less than 24 hours in duration. It is frequently related to the soak time of the gear, as drifting nets or longlines (passive gears) can be set for multiple hours as opposed to active gears, such as purse seines, where circling a school of fish can take as little as 30 minutes. One of the important results of this study was obtaining the fishing signatures for each gear type, via on-board observers, allowing us to identify fishing patterns *post-hoc* from the shape of a GPS track visualized in GIS software. Moreover, this characterization of the fishing gears enabled initial parameterization of the HMMs. In this study, two states were chosen, as this fit the bimodal distribution of boat speeds, but it would be interesting to test a model with three or four states in future studies, as some fishing techniques can be divided into four different activities: traveling, setting, soak time and hauling.

The maps showing fishing states identified by both methods (Fig 5 and Fig B in S1 File) provided insights into the idiosyncrasies of some gears. For all gears, the model predicted a few fishing steps completely outside the fishing locations, probably due to decreasing speed (which could reflect engine problems, meeting with other fishermen, etc.) that was corroborated by performance measures, which were weaker when the model tried to detect and to predict non-fishing states.

#### Surface drifting gillnet

On rare occasions, the model missed some fishing states. One of the characteristics of this fishing technique is that the boat stays attached to the net while it drifts. Occasionally, fishermen can leave the net for a few minutes (i.e. meeting with other fishermen, surveillance controls) and then, the track recorded is not the position of the fishing net but that of the boat, even if the net is still in the water.

#### Mullet circling gillnet

Here, the HMM doesn’t detect states visually interpreted as fishing by experts, mainly when the boat is close to the shore. Usually, boats decrease their speed when they approach the coast and may even stop, to rest. Interpretation errors could occur, as this gear, targeting mullets (Mugilidae) that feed on mud banks [67] in shallow water.

#### Purse seine

This fishing technique is peculiar because fishermen encircle a school of sardines and, consequently, sometimes decrease their speed to detect a school before fishing. The HMM could interpret those changes in speed as fishing. Despite those errors, it should be noted that this gear produced the best performance measures.

#### Handline

The model under-detected fishing identified by the expert. Because this fishing technique is characterized by short moves between fishing operations of short duration, it can be difficult to recognize visually and this bias could be associated with manual interpretation errors. It may be possible that models outperform visual interpretation in certain cases [68].

#### Bottom gillnet

The model under-detected fishing events compared to expert validation. Bottom gillnet fishermen have five to six different nets in the boat. They set all the nets successively (with some increases in speed between each net), wait a while, and then pull out all the nets successively. Determination of the exact position of the gear is problematic since the GPS records the boat’s track, and in this case, the boat is not attached to the gear the entire time. Experts included moves between each net as fishing time because nets were still in the water. The model identified those moves as non-fishing operation. Again, when bearing in mind the scale of the fisheries (nets extending to 1 km in length) and the fact that nets are set near each other, this difference would be negligible for a generalized view of fishing locations, however it makes a difference in time spent fishing during the trip. Due to the peculiarity of this fishing technique, a 3-state model should be tested (non-fishing, setting and hauling).

### GPS sampling interval

When the sampling interval between each location was increased, model prediction accuracy varied by gear type. For the majority of gears, accuracy decreased with the exception of bottom gillnets, where accuracy increased. Not surprisingly, accuracy increased at shorter time steps for gears with the shortest fishing operations (i.e. handlines, longlines, purse seines, sardine and mullet circling gillnets). Indeed, some fishing techniques, such as handlines can last just 10 minutes and, if the time step is larger, then a fishing operation would not be highlighted by the model. For other gears like surface drifting gillnets, the gear can remain in the water for several hours, which explains why a change of time step (between 5 seconds and 10 minutes) has little impact on the accuracy of the model.

Although it may be easy to recognize the gear employed when looking at a track from a fishing trip, it is more difficult to predict which gear will be used by a given boat type, as fishermen in this study regularly change their fishing techniques. For this reason, it would be useful to have a maximum time step to set up GPS trackers in the future. Taking into account the shortest fishing events (i.e. handlines with 10 minutes), and the requirement that a minimum of 3 points are necessary to describe a fishing event (due to angle calculations), a maximum sampling interval would be 200 seconds for the gears evaluated in this study. The best sampling interval was 120 seconds, as it produced the best accuracy and sensitivity. Based on these findings we suggest that a GPS with an autonomous battery should be set to sample locations between 120 and 200 seconds, as this will improve the model prediction accuracy as well as extend battery life during deployment.

## Conclusion

In the context of fisheries management in Gabon, utilization of HMM has allowed a considerable insight into how spatiotemporal patterns of small-scale fisheries could be assessed in the absence of VMS/AIS, and where adequate personnel and other resources cannot be guaranteed. This study provides advice to managers in such situation who would want to detect fishing events from GPS data, i.e. having a stratified sampling by gear and set up sampling time interval between 2–3 minutes. Using HMM is also a promising approach associated with a participatory data collection process. Indeed, fishermen are often interested in new technologies that allow them to map their fishing grounds, to know their real-time position and be able to check if they are, for example, within a protected area. It also allows fishers to actively demonstrate to management authorities where they actually fish. This role is particularly important when decisions are being made on fishing area closures at a governmental level. In 2014, the Gabonese government announced a program to protect at least 23% of Gabonese Exclusive Economic Zone (EEZ). Knowledge of where fishing was occurring helped the design of a new Marine Protected Area network in 2017, which finally covered 26% of the EEZ.

Finally, neighboring countries (Congo and Equatorial Guinea) have already started to deploy GPS trackers on their small-scale fisheries boats [29], following the example of the Gabonese MPA project. This labor-efficient method of analysis could be shared with those countries and any nation sharing Gabon’s characteristics and challenges.

## Supporting information

S1 File Supporting information figures and tables. (DOCX)
