# Supplementary material for: “Too Big To Ignore”: A feasibility analysis of detecting fishing events in Gabonese small-scale fisheries
Source: PLoS One. 2020 Jun 10;15(6):e0234091. doi: 10.1371/journal.pone.0234091 (PMC7286497; doi:10.1371/journal.pone.0234091)
Supplement: S1 File — (DOCX) [file pone.0234091.s001.docx]

**Figure A. Fishing signatures of A: Bottom gillnet, B: Mullet circling gillnet, C: Purse seine, D: Sardine circling gillnet, E: Longline, F: Surface drifting gillnet and, G: Handline.**


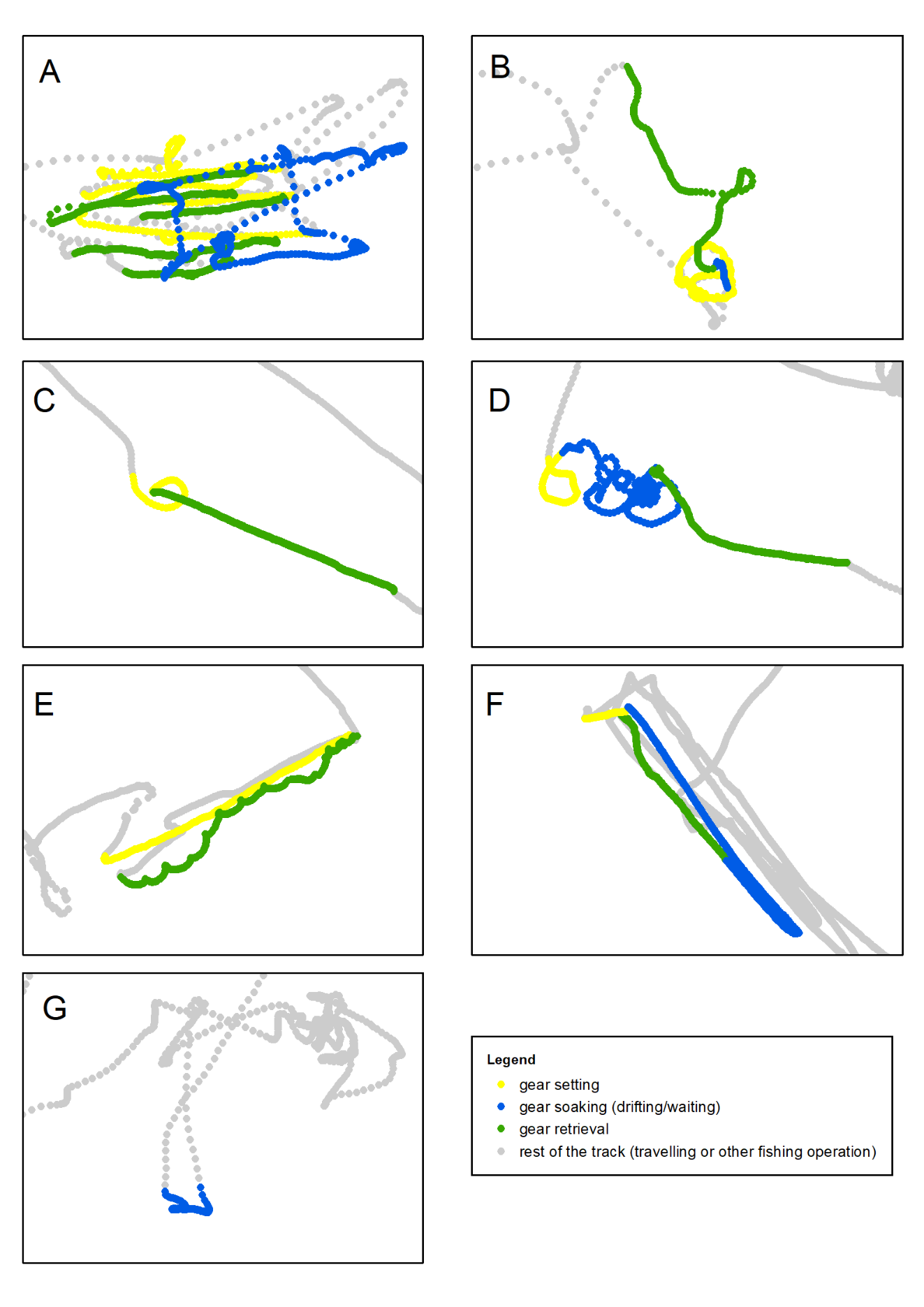


**Table A. Initial values used to fit our Hidden Markov Model.**

|  | mean step length (km) | | Standard deviation | | mean angle (radians) | | Concentration | |
| --- | --- | --- | --- | --- | --- | --- | --- | --- |
|  | Non-fishing | Fishing | Non-fishing | fishing | Non-fishing | Fishing | Non-fishing | Fishing |
| Sardine circling gillnet | 0,03626 | 0,0048969 | 0,0096529 | 0,0067565 | 0,0072246 | -0,000931 | 176,3861 | 6,36698 |
| Surface drifting gillnet | 0,0282995 | 0,0077234 | 0,0243634 | 0,0064719 | 0,0002284 | -0,000318 | 2,330935 | 8,246443 |
| Mullet circling gillnet | 0,0117012 | 0,0024814 | 0,0127724 | 0,0044001 | 0,0099217 | 0,0101323 | 2,948234 | 3,166768 |
| Purse seine | 0,0197176 | 0,0022215 | 0,0023926 | 0,0030348 | -0,001675 | -0,009571 | 252,2811 | 10,919 |
| Handline | 0,018005 | 0,0008296 | 0,0046881 | 0,0017046 | 0,0108898 | 0,1160657 | 46,89171 | 0,4794418 |
| Longline | 0,0134446 | 0,004324 | 0,0106474 | 0,0078514 | 0,0019872 | 0,0412174 | 16,27241 | 6,630557 |
| Bottom gillnet | 0,036186 | 0,0057746 | 0,0114891 | 0,0091766 | -0,004848 | -0,011173 | 130,3637 | 8,352427 |

**Figure B.** **Maps of all tracks for each gear types and fishing areas identified by visual interpretation and HMM methods (In grey: both methods predict non-fishing, in blue: both methods predict fishing, in red: only HMM predicts fishing, in green: only visual interpretation predicts fishing)**

Mullet circling gillnet


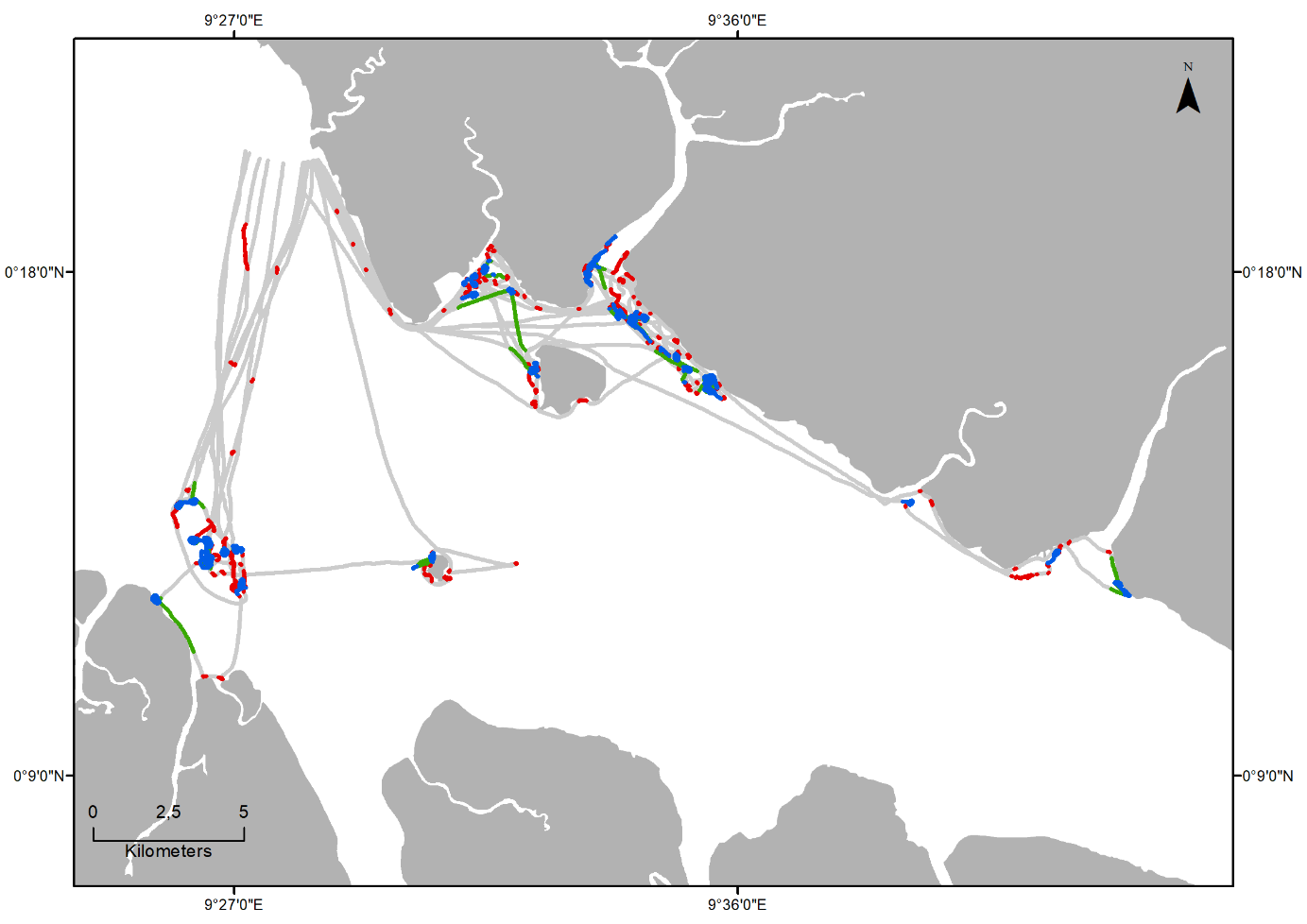


Sardine circling gillnet


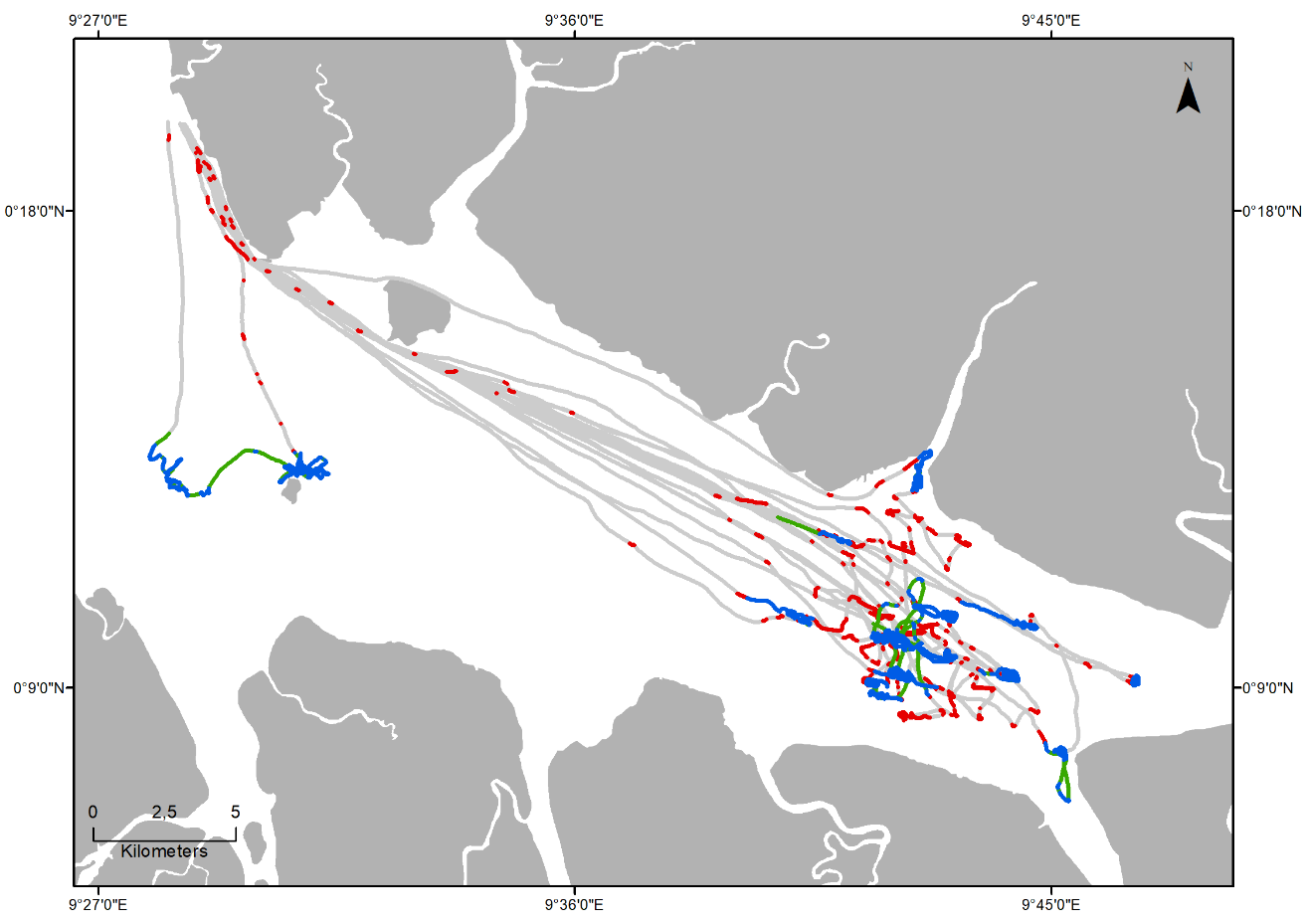


Bottom gillnet


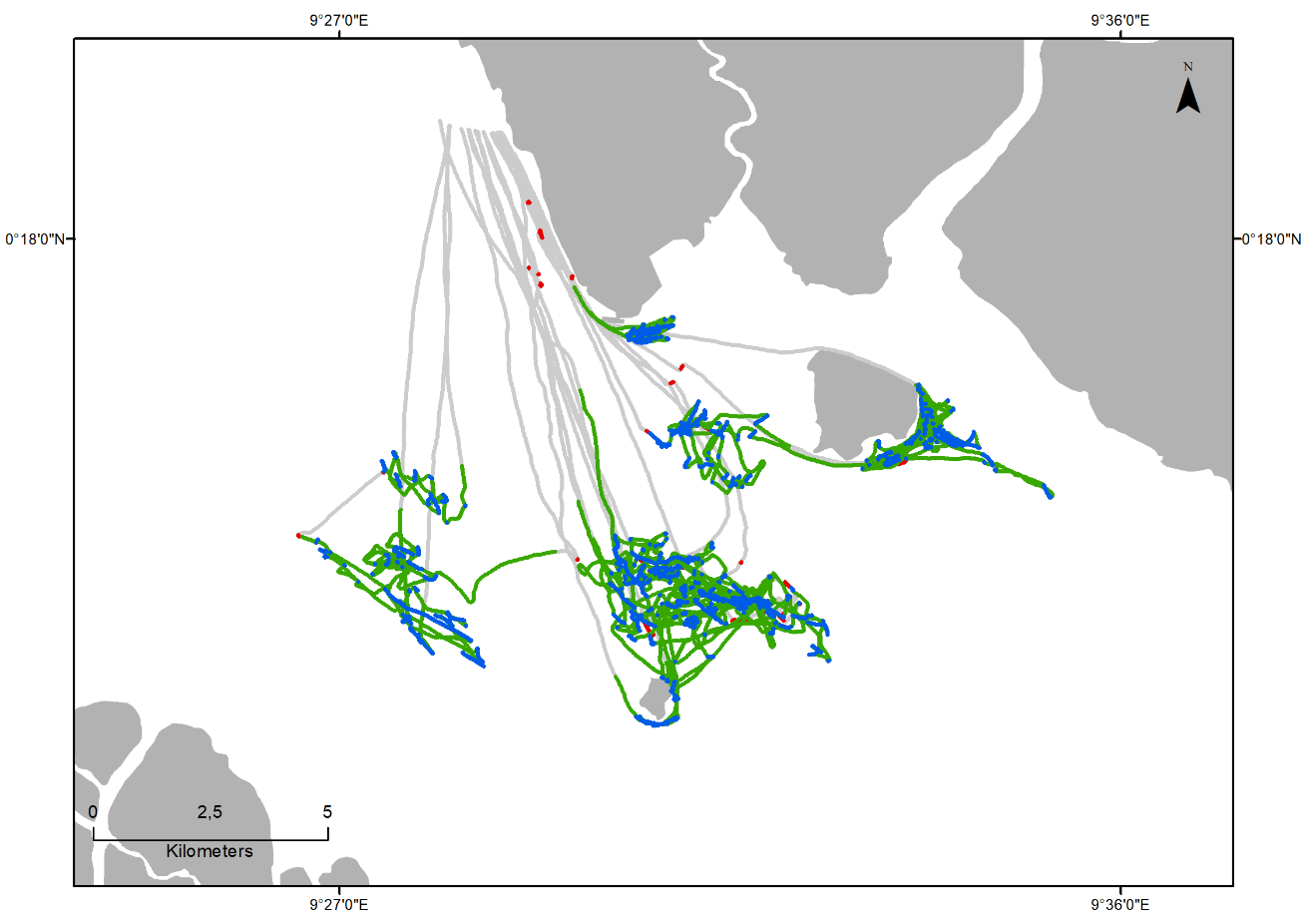


Handline


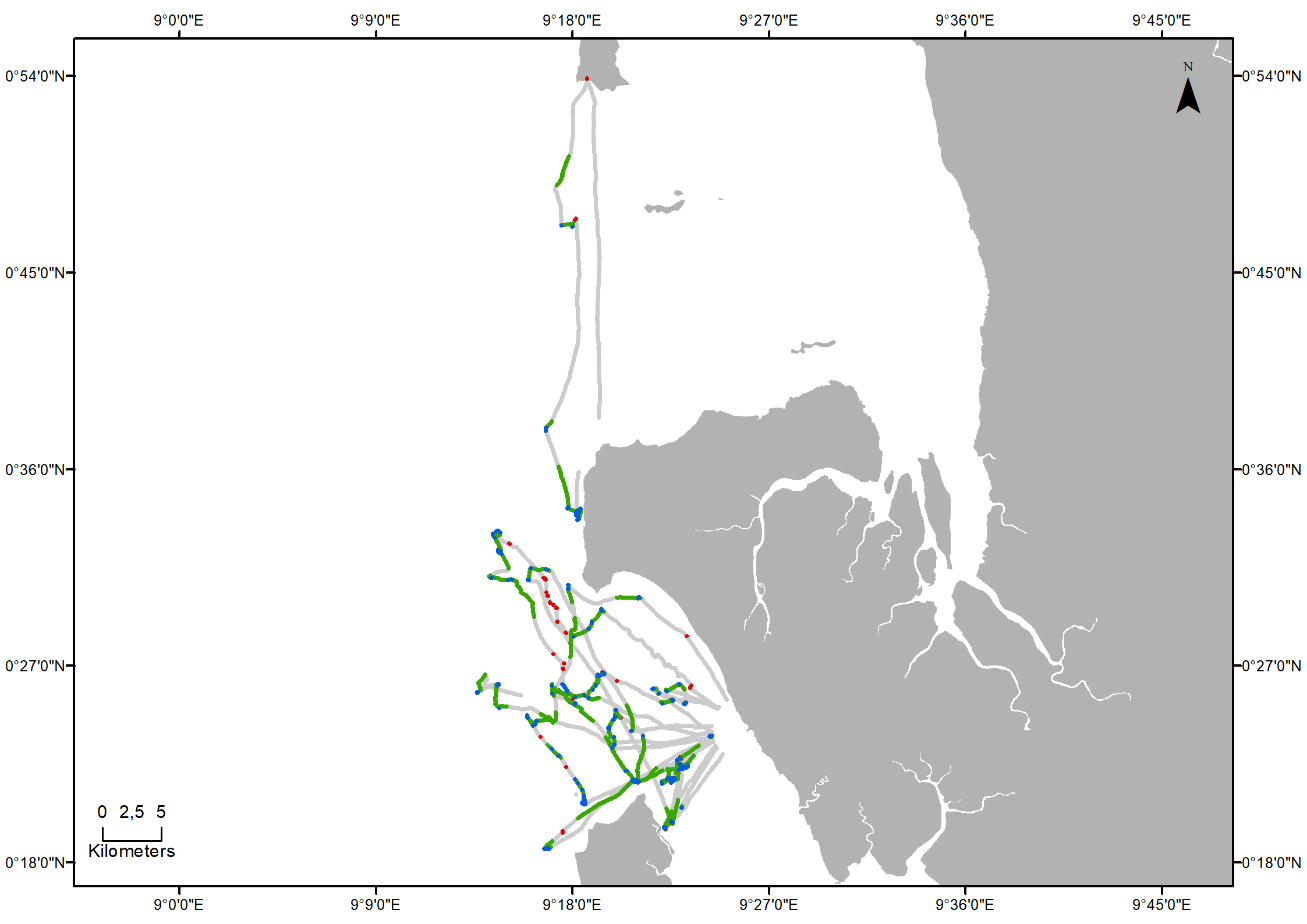


Longline


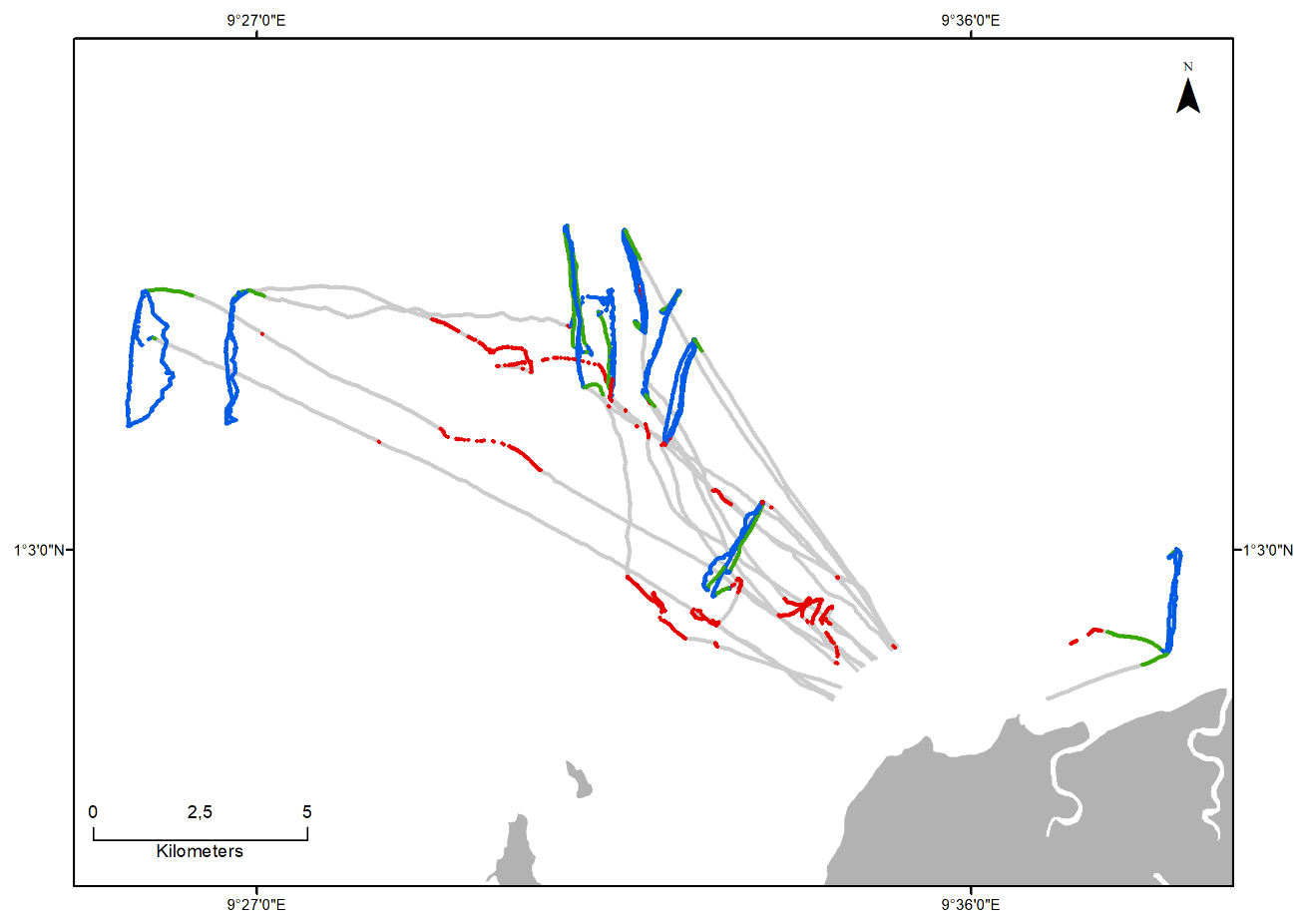


Purse seine


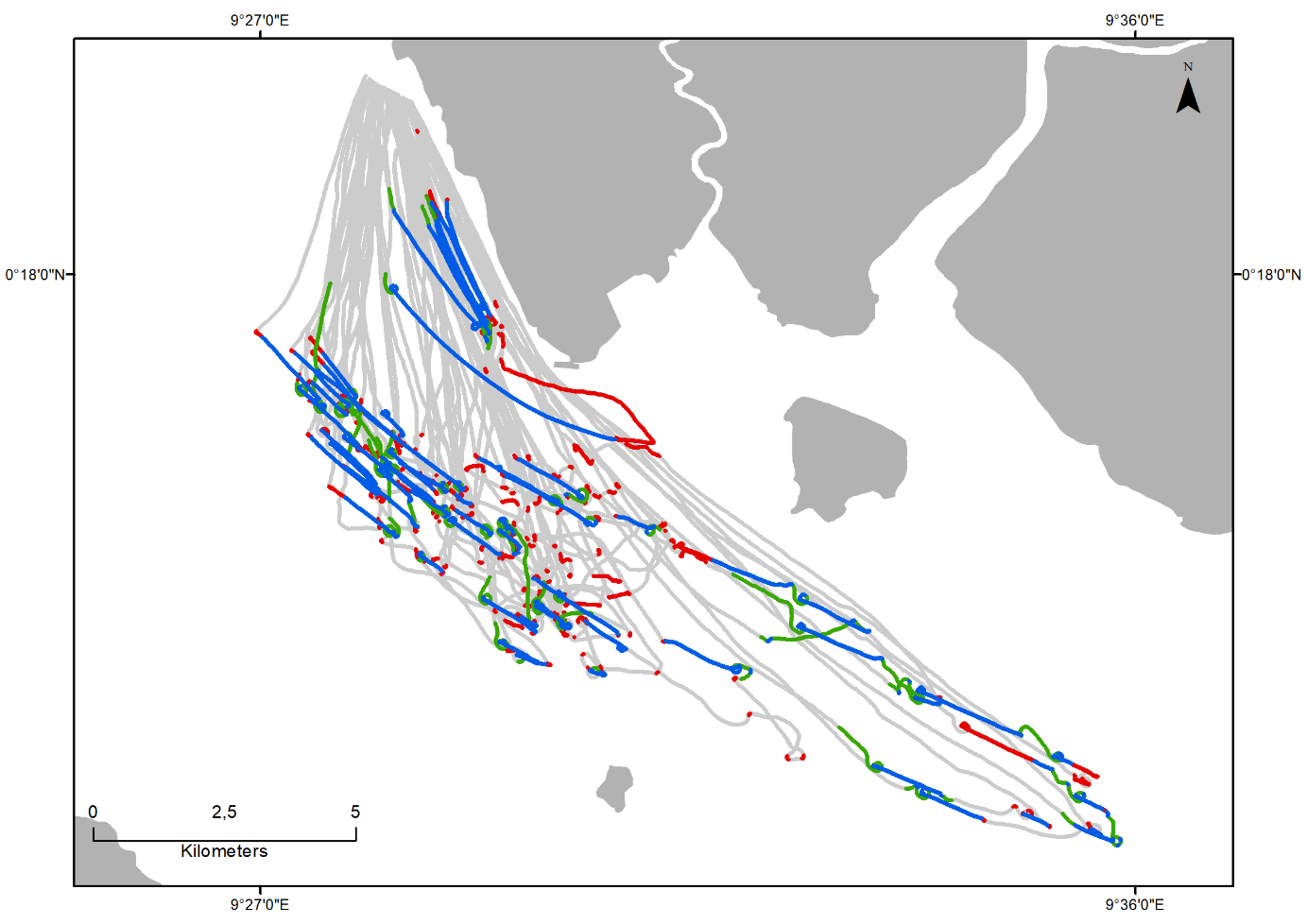


**Table B. Accuracy, sensitivity, specificity, fishing (F) prediction and non-fishing (NF) prediction from Hidden Markov Models at different time steps for each gear type (values go from 0 to 1, 1 being the best value, see methods for details)**

| Gear | Time step (sec) | Accuracy | Sensitivity | Specificity | F prediction | NF prediction |
| --- | --- | --- | --- | --- | --- | --- |
| Sardine circling gillnet | 5 | 0.78 | 0.96 | 0.6643 | 0.66 | 0.96 |
| Sardine circling gillnet | 10 | 0.78 | 0.962 | 0.6594 | 0.66 | 0.96 |
| Sardine circling gillnet | 60 | 0.77 | 0.9754 | 0.6316 | 0.64 | 0.97 |
| Sardine circling gillnet | 120 | 0.77 | 0.9889 | 0.6153 | 0.64 | 0.99 |
| Sardine circling gillnet | 180 | 0.76 | 0.989 | 0.6003 | 0.63 | 0.99 |
| Sardine circling gillnet | 240 | 0.75 | 0.9926 | 0.5789 | 0.62 | 0.99 |
| Sardine circling gillnet | 300 | 0.74 | 0.9939 | 0.5672 | 0.61 | 0.99 |
| Sardine circling gillnet | 360 | 0.74 | 0.9889 | 0.5704 | 0.61 | 0.99 |
| Sardine circling gillnet | 420 | 0.74 | 0.9915 | 0.5634 | 0.61 | 0.99 |
| Sardine circling gillnet | 480 | 0.73 | 0.9901 | 0.5537 | 0.6 | 0.99 |
| Sardine circling gillnet | 540 | 0.73 | 0.9944 | 0.5581 | 0.6 | 0.99 |
| Sardine circling gillnet | 600 | 0.72 | 0.9938 | 0.5292 | 0.59 | 0.99 |
| Sardine circling gillnet | 660 | 0.72 | 0.9797 | 0.5392 | 0.59 | 0.98 |
| Sardine circling gillnet | 720 | 0.71 | 0.9779 | 0.5404 | 0.59 | 0.97 |
| Sardine circling gillnet | 780 | 0.71 | 0.9837 | 0.5351 | 0.58 | 0.98 |
| Sardine circling gillnet | 840 | 0.7 | 0.9646 | 0.5318 | 0.57 | 0.96 |
| Sardine circling gillnet | 900 | 0.7 | 0.973 | 0.5128 | 0.59 | 0.96 |
| Surface gillnet | 5 | 0.69 | 0.76 | 0.57 | 0.76 | 0.58 |
| Surface gillnet | 10 | 0.7 | 0.7674 | 0.5816 | 0.76 | 0.59 |
| Surface gillnet | 60 | 0.7 | 0.7615 | 0.6024 | 0.71 | 0.59 |
| Surface gillnet | 120 | 0.72 | 0.7817 | 0.598 | 0.77 | 0.61 |
| Surface gillnet | 180 | 0.72 | 0.7749 | 0.6139 | 0.78 | 0.61 |
| Surface gillnet | 240 | 0.71 | 0.7653 | 0.6208 | 0.78 | 0.6 |
| Surface gillnet | 300 | 0.7 | 0.752 | 0.6178 | 0.78 | 0.59 |
| Surface gillnet | 360 | 0.7 | 0.7457 | 0.6313 | 0.78 | 0.59 |
| Surface gillnet | 420 | 0.74 | 0.7216 | 0.7687 | 0.85 | 0.61 |
| Surface gillnet | 480 | 0.73 | 0.7153 | 0.768 | 0.84 | 0.61 |
| Surface gillnet | 540 | 0.73 | 0.709 | 0.774 | 0.85 | 0.6 |
| Surface gillnet | 600 | 0.73 | 0.6977 | 0.7798 | 0.85 | 0.59 |
| Surface gillnet | 660 | 0.73 | 0.6977 | 0.7798 | 0.85 | 0.59 |
| Surface gillnet | 720 | 0.73 | 0.6977 | 0.7798 | 0.85 | 0.59 |
| Surface gillnet | 780 | 0.68 | 0.697 | 0.6494 | 0.78 | 0.55 |
| Surface gillnet | 840 | 0.68 | 0.7006 | 0.6438 | 0.78 | 0.55 |
| Surface gillnet | 900 | 0.68 | 0.7 | 0.643 | 0.78 | 0.55 |
| Mullet circling gillnet | 5 | 0.75 | 0.95 | 0.57 | 0.67 | 0.92 |
| Mullet circling gillnet | 10 | 0.75 | 0.9667 | 0.5497 | 0.67 | 0.95 |
| Mullet circling gillnet | 60 | 0.74 | 0.9795 | 0.5208 | 0.66 | 0.96 |
| Mullet circling gillnet | 120 | 0.73 | 0.9854 | 0.5017 | 0.65 | 0.97 |
| Mullet circling gillnet | 180 | 0.74 | 0.982 | 0.5265 | 0.65 | 0.97 |
| Mullet circling gillnet | 240 | 0.76 | 0.9818 | 0.5605 | 0.68 | 0.97 |
| Mullet circling gillnet | 300 | 0.76 | 0.9817 | 0.5572 | 0.67 | 0.97 |
| Mullet circling gillnet | 360 | 0.77 | 0.9778 | 0.5804 | 0.68 | 0.97 |
| Mullet circling gillnet | 420 | 0.76 | 0.9714 | 0.5522 | 0.67 | 0.95 |
| Mullet circling gillnet | 480 | 0.76 | 0.9676 | 0.5517 | 0.67 | 0.95 |
| Mullet circling gillnet | 540 | 0.74 | 0.9835 | 0.5247 | 0.66 | 0.97 |
| Mullet circling gillnet | 600 | 0.72 | 0.9863 | 0.4788 | 0.64 | 0.97 |
| Mullet circling gillnet | 660 | 0.72 | 0.9799 | 0.472 | 0.63 | 0.96 |
| Mullet circling gillnet | 720 | 0.71 | 0.9669 | 0.4747 | 0.63 | 0.94 |
| Mullet circling gillnet | 780 | 0.7 | 0.994 | 0.44348 | 0.61 | 0.99 |
| Mullet circling gillnet | 840 | 0.69 | 0.974 | 0.4386 | 0.61 | 0.95 |
| Mullet circling gillnet | 900 | 0.68 | 0.9784 | 0.4207 | 0.59 | 0.96 |
| Purse seine | 5 | 0.85 | 0.94 | 0.77 | 0.79 | 0.94 |
| Purse seine | 10 | 0.85 | 0.969 | 0.7437 | 0.77 | 0.96 |
| Purse seine | 60 | 0.83 | 0.9798 | 0.6938 | 0.74 | 0.97 |
| Purse seine | 120 | 0.81 | 0.9856 | 0.6475 | 0.72 | 0.98 |
| Purse seine | 180 | 0.75 | 0.9889 | 0.5337 | 0.66 | 0.98 |
| Purse seine | 240 | 0.74 | 0.9979 | 0.5106 | 0.65 | 1 |
| Purse seine | 300 | 0.74 | 0.996 | 0.5 | 0.64 | 0.99 |
| Purse seine | 360 | 0.72 | 0.9968 | 0.4791 | 0.63 | 0.99 |
| Purse seine | 420 | 0.72 | 0.9944 | 0.4824 | 0.63 | 0.99 |
| Purse seine | 480 | 0.71 | 0.9935 | 0.4646 | 0.62 | 0.99 |
| Purse seine | 540 | 0.71 | 0.9928 | 0.4557 | 0.62 | 0.99 |
| Purse seine | 600 | 0.69 | 0.9947 | 0.4207 | 0.61 | 0.99 |
| Purse seine | 660 | 0.7 | 0.9971 | 0.4316 | 0.61 | 0.99 |
| Purse seine | 720 | 0.69 | 1 | 0.42 | 0.6 | 1 |
| Purse seine | 780 | 0.69 | 0.9965 | 0.4188 | 0.61 | 0.99 |
| Purse seine | 840 | 0.67 | 1 | 0.3907 | 0.59 | 1 |
| Purse seine | 900 | 0.68 | 0.996 | 0.3863 | 0.59 | 0.99 |
| Hand line | 5 | 0.74 | 0.85 | 0.43 | 0.8 | 0.51 |
| Hand line | 10 | 0.73 | 0.8333 | 0.4439 | 0.8 | 0.5 |
| Hand line | 60 | 0.71 | 0.7902 | 0.4774 | 0.8 | 0.46 |
| Hand line | 120 | 0.7 | 0.787 | 0.4746 | 0.8 | 0.45 |
| Hand line | 180 | 0.7 | 0.7755 | 0.4916 | 0.81 | 0.45 |
| Hand line | 240 | 0.68 | 0.7533 | 0.4918 | 0.8 | 0.42 |
| Hand line | 300 | 0.68 | 0.7419 | 0.5185 | 0.81 | 0.43 |
| Hand line | 360 | 0.67 | 0.7232 | 0.5156 | 0.8 | 0.41 |
| Hand line | 420 | 0.67 | 0.7332 | 0.5156 | 0.8 | 0.41 |
| Hand line | 480 | 0.65 | 0.7016 | 0.5165 | 0.8 | 0.39 |
| Hand line | 540 | 0.65 | 0.685 | 0.5427 | 0.81 | 0.38 |
| Hand line | 600 | 0.64 | 0.679 | 0.5338 | 0.8 | 0.38 |
| Hand line | 660 | 0.64 | 0.6707 | 0.5661 | 0.81 | 0.38 |
| Hand line | 720 | 0.62 | 0.6361 | 0.5928 | 0.81 | 0.37 |
| Hand line | 780 | 0.62 | 0.6295 | 0.5829 | 0.8 | 0.37 |
| Hand line | 840 | 0.61 | 0.6207 | 0.5812 | 0.8 | 0.36 |
| Hand line | 900 | 0.6 | 0.6086 | 0.5932 | 0.8 | 0.35 |
| Long line | 5 | 0.75 | 0.96 | 0.43 | 0.72 | 0.88 |
| Long line | 10 | 0.75 | 0.9651 | 0.4343 | 0.72 | 0.89 |
| Long line | 60 | 0.75 | 0.9716 | 0.4269 | 0.72 | 0.91 |
| Long line | 120 | 0.7 | 0.8484 | 0.474 | 0.71 | 0.67 |
| Long line | 180 | 0.66 | 0.7908 | 0.4655 | 0.69 | 0.6 |
| Long line | 240 | 0.64 | 0.6654 | 0.5908 | 0.71 | 0.54 |
| Long line | 300 | 0.64 | 0.5529 | 0.7734 | 0.79 | 0.54 |
| Long line | 360 | 0.62 | 0.5447 | 0.7359 | 0.76 | 0.52 |
| Long line | 420 | 0.63 | 0.5709 | 0.72 | 0.75 | 0.53 |
| Long line | 480 | 0.64 | 0.5192 | 0.815 | 0.81 | 0.53 |
| Long line | 540 | 0.64 | 0.5259 | 0.8182 | 0.81 | 0.53 |
| Long line | 600 | 0.64 | 0.6029 | 0.7029 | 0.75 | 0.54 |
| Long line | 660 | 0.64 | 0.5474 | 0.792 | 0.8 | 0.54 |
| Long line | 720 | 0.64 | 0.5872 | 0.7179 | 0.75 | 0.54 |
| Long line | 780 | 0.62 | 0.5312 | 0.757 | 0.77 | 0.52 |
| Long line | 840 | 0.58 | 0.3108 | 0.98 | 0.96 | 0.49 |
| Long line | 900 | 0.5 | 0.1727 | 0.9785 | 0.93 | 0.44 |
| Bottom gillnet | 5 | 0.75 | 0.75 | 0.75 | 0.94 | 0.36 |
| Bottom gillnet | 10 | 0.85 | 0.8819 | 0.6865 | 0.94 | 0.52 |
| Bottom gillnet | 60 | 0.9 | 0.9453 | 0.66 | 0.94 | 0.69 |
| Bottom gillnet | 120 | 0.92 | 0.9697 | 0.6399 | 0.94 | 0.8 |
| Bottom gillnet | 180 | 0.93 | 0.9762 | 0.6527 | 0.94 | 0.83 |
| Bottom gillnet | 240 | 0.93 | 0.9784 | 0.6557 | 0.94 | 0.85 |
| Bottom gillnet | 300 | 0.93 | 0.9872 | 0.6319 | 0.94 | 0.9 |
| Bottom gillnet | 360 | 0.93 | 0.9908 | 0.625 | 0.93 | 0.94 |
| Bottom gillnet | 420 | 0.94 | 0.991 | 0.6408 | 0.94 | 0.93 |
| Bottom gillnet | 480 | 0.93 | 0.9938 | 0.5934 | 0.93 | 0.95 |
| Bottom gillnet | 540 | 0.93 | 0.9938 | 0.5934 | 0.93 | 0.95 |
| Bottom gillnet | 600 | 0.93 | 0.9938 | 0.5934 | 0.93 | 0.95 |
| Bottom gillnet | 660 | 0.93 | 0.9938 | 0.5934 | 0.93 | 0.95 |
| Bottom gillnet | 720 | 0.93 | 0.9938 | 0.5934 | 0.93 | 0.95 |
| Bottom gillnet | 780 | 0.93 | 0.9938 | 0.5934 | 0.93 | 0.95 |
| Bottom gillnet | 840 | 0.93 | 0.9938 | 0.5934 | 0.93 | 0.95 |
| Bottom gillnet | 900 | 0.93 | 0.9938 | 0.5934 | 0.93 | 0.95 |
